# New Method of Preserving Organic Matters

**Published:** 1846-10

**Authors:** 


*New Method of Preserving Organic Matters.*—At a late soirêe, held by the Marquis of Northampton, Dr. Silvestri, physician to the Royal Hospital at Naples, and chief physician of his majesty's guard of honor, exhibited several preparations made according to a method discovered by him. By this process organic matters are perfectly preserved, being converted into a substance possessing the hardness of stone, and admitting of being polished. Among the preparations shown, were a portion of human liver, a section of a kidney, a section of a testis, and some hands and heads. In these specimens the texture was perfectly apparent when examined with a magnifying-glass, but the substances themselves had acquired the hardness and resonance of stone. The head oi a ram possessed the stony hardness peculiar to these preparations, while the ears and hairs retained their natural softness and pliability. Birds, submitted to this process, retain their feathers uninjured either in color or pliancy; fishes are coated by a kind of transparent varnish; and the cornea retains the transparency of life. Dr. Silvestri has also succeeded in preserving flowers in the same manner, the petals retaining their natural hues, and the stem and leaves their pliancy and verdure. He gives the following statement of the applications of his discovery:

An entire corpse, without being injured in the slightest degree, can be brought to a consistency appreoaching to petrifaction, and preserved for an infinite period in full perfection of form, with the hair, nails, &c. Like a statute, it can also be placed in any given position, as illustrative of individual character or station.

Animals of every species, from the elephant to the insect, are susceptible of being reduced to the same state of consistency and preservation. The plumage, fur, wool, and all other adjuncts of nature, remain entire, retaining the same color, firmness, and flexibility that they had at the moment of death.

The same result can be produced in the single parts, organs, &c., of any organic animal body, without undergoing any alteration whatever, even though injected previously to the operation.

All the objects in question may be petrified in such various degrees of intensity as may be judged necessary for the purposes of dissection, observation, examination, &c., with perfect freedom from stench, and all else of an objectionable nature, either when handled, or preserved as objects of curiosity.

Flowers and plants can be preserved unchangeable, with their colors, form, leaves, and stems, as if just gathered.—*Lancet.—Medical News.*

